# Congenital Renal Arteriovenous Malformation: Diagnostic Clues and Methods

**DOI:** 10.3390/medicina57121304

**Published:** 2021-11-28

**Authors:** Seung-Kwon Choi, Gyeong Eun Min, Dong-Gi Lee

**Affiliations:** 1 Department of Urology, Seoul Medical Center, Seoul 02053, Korea; urocsk0127@hanmail.net; 2 Department of Urology, School of Medicine, Kyung Hee University, Seoul 05278, Korea; danielmin73@gmail.com

**Keywords:** arteriovenous malformation, hematuria, kidney

## Abstract

*Background and objectives:* Renal arteriovenous malformation (AVM) is a rare disease and is difficult to be diagnosed by conventional methods because of its rarity. In this study, we investigated the diagnostic clues, and made an algorithm for the better diagnosis of renal AVM. *Materials and Methods:* We reviewed 13 patients who were diagnosed with AVM by using renal angiography from 1986 to 2020 at our institutes. We evaluated clinical features, diagnostic tools, treatment modalities, and outcomes after the treatment of patients. *Results:* All patients were female, and the mean age was 36.9 years (range 19 to 54 years). Twelve (92.3%) patients complained of gross hematuria. Four (30.8%) patients showed symptoms in relation with pregnancy and delivery. Angiographic findings demonstrated cirsoid type in 10 patients and aneurysmal type in 3 patients. Among the 11 patients who underwent computed tomography, AVMs were detected in 3 (27.3%) patients. Renal duplex Doppler was performed in 6 patients, and all of these patients were diagnosed with AVM, demonstrating a vascular turbulence or blood-rich area. Twelve patients were initially treated with transarterial embolization. Nephrectomy was performed in two patients due to persistent bleeding with hypovolemic shock. *Conclusions:* We should consider possible AVMs in patients who were not detected by conventional work up for hematuria, especially in mid-aged, pregnant, or recently delivered women. Renal duplex Doppler might be the optimal diagnostic modality in these patients. Our diagnostic algorithm could be aid to diagnosis and treatment for renal AVM patients.

## 1. Introduction

Renal arteriovenous malformation (AVM) is an aberrant communication between the renal arterial and venous systems in the kidney. AVMs are rare disease entities, occurring in less than 1% of the general population (fewer than 200 cases in the literature) [1]. Most AVMs are not detected until adulthood and are primarily congenital. The clinical manifestations of AVM vary from asymptomatic presentation, hematuria, flank pain, perinephric hematoma, abdominal mass, abdominal bruit, and hypertension to high output heart failure [1]. Usually, patients are presented with hematuria or found incidentally at diagnostic imaging. AVMs are classified into three types as follows: cirsoid, angiomatous, and aneurysmal types. The cirsoid AVM is the most common type. It appears as multiple dilated feeding arteries and draining veins with a knotted, tortuous appearance of numerous vessels and multiple arteriovenous interconnections [2,3]. Angiomatous AVM consists of a single large artery feeding multiple interconnecting distal branches and draining veins [2,3]. Aneurysmal or idiopathic AVMs are composed of a single feeding artery and a single draining vein with aneurysmal dilatation [1]. Conservative treatment (observation or surveillance) can be initiated in patients with asymptomatic AVM, whereas in cases of uncontrolled hematuria, hypovolemic shock, hypertension, heart failure, or rupture, treatments such as embolization or surgery should be considered, regardless of AVM type. Because AVMs are rare and present with hematuria, it is important to differentiate AVM from other renal lesions that present with hematuria. However, AVMs, especially the small cirsoid type, are difficult to diagnose by conventional methods such as intravenous pyelography, computed tomography (CT), and gray scale ultrasonography (US). Thus, the diagnosis of AVM is often delayed. Furthermore, because of their rare disease entities, there are no diagnostic and therapeutic guidelines regarding AVMs.

In this study, we investigated the diagnostic clues and methods for AVMs to aiding diagnosis of patients who might confuse them with other disease. Furthermore, we determined the optimal diagnosis and treatment strategy for AVMs based on our findings.

## 2. Materials and Methods

From our Institutional Review Board approved database, we reviewed AVM patients who were diagnosed with AVMs by renal angiography in our institute from 1986 to 2020. Patients with acquired arteriovenous fistula and/or patients who had history of iatrogenic renal surgery, procedure, traumatic renal injury, and disease were excluded. After exclusion of those patients, 13 patients comprised the final cohort. The study was conducted in accordance with the guidelines of the Declaration of Helsinki and approved by the institutional review board of Kyung Hee University Hospital at Gangdong (IRB number: KHNMC 2018-07-018).

The non-invasive imaging studies for evaluation of AVMs were intravenous pyelography, kidney US, color Doppler US, and contrast-enhanced multi-detector abdominal CT (0.3 and 0.5 mm slice thickness).

When patients with hematuria visit our institutes, we routinely obtain their medical history and perform physical examination to assess the risk for genitourinary malignancy, medical renal disease, or other disease. Following initial evaluation, we stratify the risk of patients with hematuria for genitourinary malignancy or other disease. Then, we usually perform CT, conventional US, or cystoscopy for the differential diagnosis of genitourinary malignancy or other disease entities in these patients. In patients with suspicious lesions in the kidney or persistent hematuria despite negative results, we performed color Doppler US and/or renal angiography.

In this study, all patients received renal angiography for diagnosis and confirmation of AVMs. If AVM lesion had been found, initial therapeutic angio-embolization was tried. Using the Seldinger technique, cobraside catheter was placed in the main renal artery to obtain baseline selective angiography. For superselection of lesions, microcatheter was placed in feeding artery of AVMs. Embolic materials were 99% ethanol with lipiodol, absorbable gelatine sponge or Polyvinyl alcohol (PVA) particle (Contour, 150–250 µm). Patients who failed to super-select the feeding vessels underwent angio-embolization at the level of segmental renal arteries.

We reviewed the clinical features (age, sex, symptom), diagnostic modalities, angiographic findings (site and type of AVM lesion), treatment modalities, and treatment outcomes of patients. Based on characteristics of patients, we developed a diagnostic algorithm for clinical implication of our findings.

All the variables, which included the categorical and continuous ones, were used for the descriptive analyses. All the analyses were performed using SPSS version 18.0. (SPSS Inc., Chicago, IL, USA).

## 3. Results

All patients were female and the mean age was 36.9 years (range 19 to 54 years). Twelve patients (92.3%) initially presented with gross hematuria. Of the 12 patients, 8 (66.7%) patients previously experienced recurrent gross hematuria and spontaneous resolution. Only one patient showed microscopic hematuria in urinalysis for routine health check-up (Table 1).

Four (30.8%) patients showed symptoms in relation with pregnancy and delivery. Specifically, two patients developed symptoms after child delivery, and two patients developed symptoms during the pregnancy. Nine (69.2%) patients appealed flank pain. Physical examination revealed no abdominal bruits in all patients. All patients had no past medical history such as hypertension. Almost all patients showed stable initial vital signs, but one patient showed hemodynamically unstable vital signs with hypovolemic shock due to hemorrhage from AVM.

Eleven (84.6%) patients underwent CT scan for the diagnosis of the lesion which caused hematuria. Among them, two (18.2%) patients showed mass-like vascular lesions and were diagnosed with aneurismal AVM, and one (9.1%) patient showed suspicious tortuous dilated vascular structure in the kidney. However, the remaining eight (72.7%) patients showed hydronephrosis caused by blood clots in the renal pelvis and failed to detect other vascular abnormalities.

**Figure 1:**
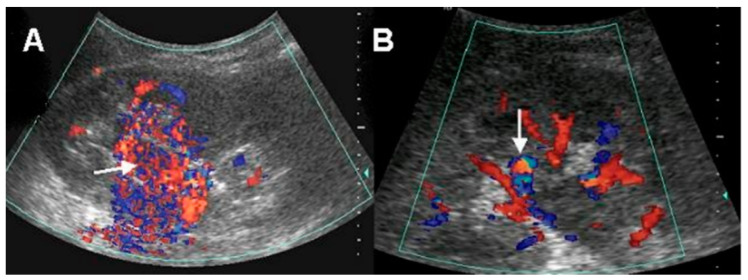
Doppler ultrasonographic findings: (**A**) conglomerated vascular structure (arrow) with blood-flow-rich area, (**B**) vascular turbulence (arrow).

Six (46.2%) patients underwent renal duplex color Doppler US. All of these patients showed a high velocity of blood flow in the renal parenchyma and were correctly diagnosed as AVM. Of them, two (33.3%) patients showed vascular mass-like lesions, and four (66.7%) patients showed a vascular turbulence lesion. (Figure 1).

**Figure 2:**
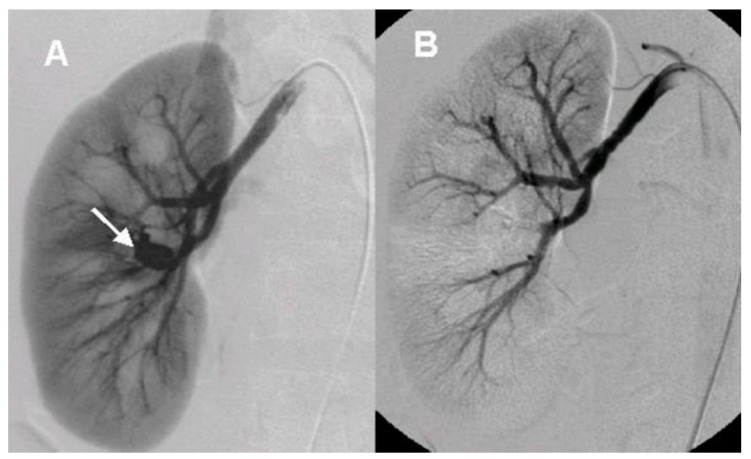
The angiographic findings of idiopathic arteriovenous malformation (**A**). Renal angiography shows aneurysmal deformity (arrow) with early venous drainage at mid-upper portion (**B**). Anterosuperior segmental artery was embolized with mixture of 99% ethanol and lipiodol 2 mL.

**Figure 3:**
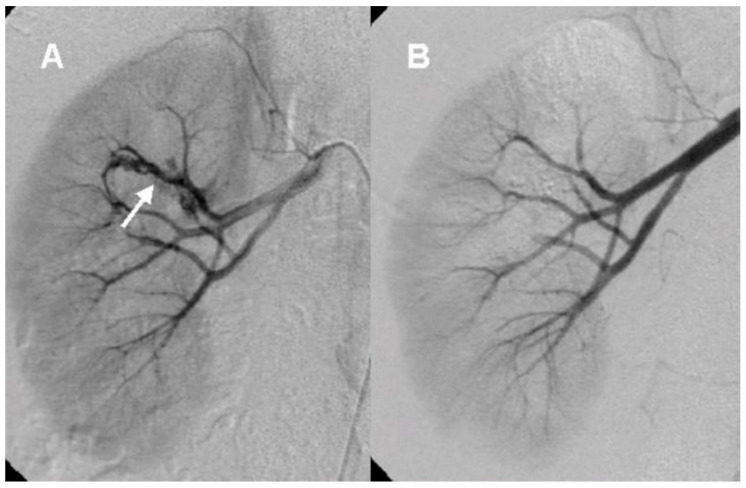
The angiographic findings of cirsoid arteriovenous malformation (**A**). Renal angiography shows tangled and conglomerated vascular structure (arrow, cirsoid type) with early venous drainage (**B**). Embolization was performed with PVA particle (Contour, 150–250 µm).

After the initial lab test and imaging study, all patients underwent diagnostic renal angiography for suspicious signs or symptoms such as persistent hematuria, flank pain, hydronephrosis, hematoma in renal pelvis, or hematuria from ureteral orifice on cystoscopy. Angiographic findings showed tortuous vascular communications or aneurysmal dilation with early venous drainage (Figure 2 and Figure 3).

Among the total of 13 patients, 10 (76.9%) patients showed cirsoid type and 3 (23.1%) patients were aneurysmal (idiopathic) type. AVM lesions were located in the right kidney in eight (61.5%) patients, the left kidney in four (30.8%) patients, and both kidneys in one (7.7%) patient. Multiple AVM lesions were observed in the same kidney in one patient. No abnormal vascular lesion was observed in the contralateral renal angiography, except for the patient who had AVM in both sides.

Twelve (92.3%) patients were initially treated with transarterial embolization (TAE) for AVM. Among them, one patient (patient No. 5) presented with persistent hematuria and signs of hypovolemic shock after TAE, demonstrating success rate after the initial TAE of 91.7%. She received urgent nephrectomy for clinical failure of TAE. TAE was not attempted in one patient (patient No. 4) for risk of total renal ischemia due to the AVM involving multiple large renal arteries. She underwent nephrectomy for initial treatment of large multiple AVM. Despite no medical history of hypertension, one patient showed high blood pressure at visit to our institute. However, her blood pressure was normalized after treatment. The remaining 12 patients showed normal blood pressure before and after treatment. Any sign of heart failure or renal insufficiency was not observed before and after treatment in follow up periods.

## 4. Discussion

Renal AVM is a complex network with tortuous dilated vessels and multiple abnormal arteriovenous communications via a vascular nidus, and usually involves feeding arteries at the segmental and interlobar level [4]. Renal AVMs involve dysplastic subepithelial vessels with the absence of elastic lamina and located beneath the mucosa of renal calyx or renal pelvis. Thus, they cause hematuria, massive hemorrhage, flank pain, hypertension, and congestive heart failure [3,5,6]. Although renal AVM is an uncommon cause of hematuria because of its rarity, hematuria is the most common presenting symptom of renal AVM patients due to rupture of small venules into the renal collecting system from increased intravascular pressure [4]. Therefore, it is important to differentiate AVM from other lesions that cause hematuria such as renal cell carcinoma, ureter stone, inflammation, and other vascular abnormalities. There are multiple imaging modalities to demonstrate renal AVM such as intravenous pyelography, CT, and US. Intravenous pyelography usually shows a hydronephrosis with filling defects in the renal pelvis, suggesting urothelial tumor or hematoma. Gray-scale US reflects hypoechoic cystic or tubular-like structures of varying sizes [1]. The CT scan of renal AVM demonstrates mass-like lesions with vascular density and dilated draining renal veins.

In clinical practice, physicians usually perform CT or conventional US for differential diagnosis of disease entities such as renal tumor or other causative lesion in patients with hematuria. However, these noninvasive diagnostic methods are not enough to detect all of AVM lesion. In some cases, there is no demonstrable vascular lesion. Hematoma in the renal pelvis might be the only clue on CT or conventional US. Therefore, renal AVM is a diagnostic and therapeutic challenge in clinical practice. Eom et al. assessed the safety and efficacy of TAE in renal AVM patients [7]. They reported that among patients who underwent CT, 72% of patients were given the correct diagnosis, 23% showed hematoma in renal pelvis with hydronephrosis, and in 5% of patients it was misdiagnosed as an aneurysm. In the case of US, 70% of patients received the correct diagnosis, 20% failed to detect any abnormality, and in 10% of patients it was misdiagnosed as an aneurysm. Another study reported that 50% of patients with AVM were confirmed by CT, but no definite diagnosis could be made for the remaining patients because no abnormal lesion was found on CT [8]. Aneurysmal AVMs are often detected by CT scan because they are larger than 1 cm in diameter and located near the renal hilum. However, cirsoids, especially small cirsoid types, are difficult to diagnose by CT [1,9]. In the current study, 27.3% of the patients were diagnosed with AVM or suspicious vascular abnormality by CT, while the remaining 72.7% of patients showed hydronephrosis with blood clot in renal pelvis and failed to detect AVM.

The duplex color Doppler US in AVM demonstrates multidirectional, turbulent, and increased flow velocity, a mosaic pattern, and perivascular soft tissue color speckling [1,10]. Spectral analysis showed increased velocity and decreased resistance [1,11]. High-velocity blood flow with a diastolic component in the feeding artery and arterial pulsations in the draining vein is characteristic of arteriovenous shunting. Nagamura et al. revealed that color Doppler US in AVM clearly showed an area rich in blood flow with innumerable posterior color spots [12]. Several studies have suggested that duplex color Doppler US is useful in assessment of renal AVM including small cirsoid type and for the differential diagnosis of other mimicking disease entities [10,11,12,13]. Takebayashi et al. assessed color Doppler US in six patients with AVMs. In their study, AVMs were detected with focal areas of flow and as a mixing of lighter colors. They suggested that color Doppler US can be used for diagnosing and monitoring AVMs and could eliminate a considerable number of angiograms [10]. Donmez et al. suggested that duplex color Doppler US provides more information than gray-scale US for the diagnosis of renal AVM with cystic lesions showing the vascularization of the septa or the solid component of the cystic lesion [13]. Using duplex color Doppler US, small renal AVMs that are not visible by conventional US can be detected. We confirmed those findings in this study. In our results, patients who underwent renal duplex color Doppler US were all correctly diagnosed as AVM. Despite its invasiveness, arteriography is the gold standard for evaluating renal AVM. Taken together, we consider duplex color Doppler US as useful non-invasive diagnostic method for renal AVM, and it would be recommended in patients with no apparent cause of hematuria on conventional US or CT.

**Figure 4:**
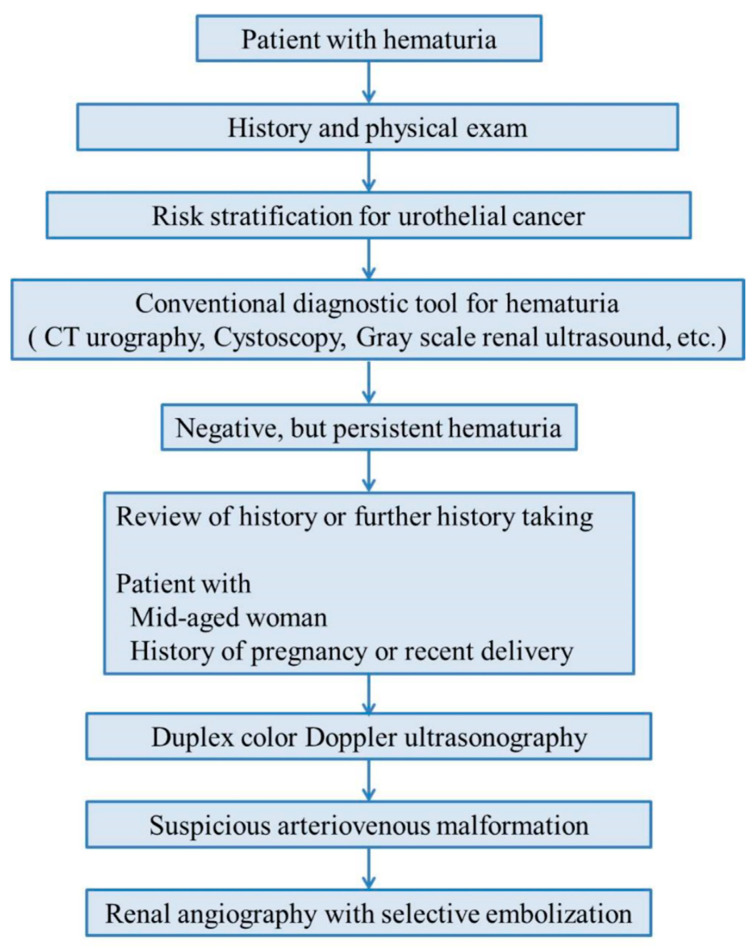
The diagnostic clues for renal arteriovenous malformation.

In the current study, we confirmed the following characteristics of renal AVM described in previous studies. Almost all patients presented with gross hematuria (12 patients, 92.3%). The most common type of AVM was cirsoid (10 patients, 76.9%). The right kidney was more frequently involved than the left (right: 9 patients, 69.2%) [14]. All patients were women. Congenital renal AVM is a female-predominant disease according to the published case reports, though there are a few reports of AVM in men [8,15]. The age of patients was from 30 to 40 years old, demonstrating that their mean age was 36.9 years [16]. Four (30.8%) patients were related with pregnancy [16]. TAE is a safe and effective treatment of renal AVM demonstrating a success rate of 91.7% in our results [7]. Based on our findings and diagnostic clues, we developed an algorithm for the diagnosis of renal AVM (Figure 4).

According to our diagnostic clues, hematuria in 30–40-year-old woman with history of recent pregnancy or delivery, and no apparent cause of hematuria on conventional imaging study is strongly suggestive of renal AVM. Gross hematuria from the ureteral orifice may also be a clue for renal AVM during cystoscopy. Furthermore, duplex color Doppler US is an important non-invasive diagnostic tool for renal AVM.

Some limitations of this study should be noted, including the potential bias inherent in retrospective studies. Our study cohort was small. However, considering AVM is a rare disease entity, the number of patients is relatively not small for two institutes. Despite these limitations, our findings and diagnostic algorithm will be useful not only for differentiate AVM patients with other similar disease entities but also useful for counseling and treatment decision making in these patients.

## 5. Conclusions

Renal AVM is a rare disease that causes hematuria, and it is a diagnostic challenge in clinical practice. Our diagnostic algorithm of renal AVM can be guided to correct diagnosis and treatment decisions for possible renal AVM patients. When the patient is a mid-aged woman, when the patient has the experiences of recent delivery or pregnancy, when the patient complains of recurrent gross hematuria, and when no definite diagnosis could be made with conventional methods, we strongly consider the possibility of renal AVM. Renal duplex color Doppler is an effective non-invasive method for the diagnosis of AVMs. Therefore, when CT or conventional US find no detectable causative lesion in those patients, renal duplex color Doppler US should be considered immediately.

## Figures and Tables

**Table 1:** Patient characteristics of renal arteriovenous malformations.

Case	Age/Sex	Main Complaint	Hypertension	Pregnancy	Site	Type	Initial Treatment	Recurrence
1	54/F	gross hematuria	+	-	Right mid pole, Left upper pole	cirsoid	embolization	-
2	16/F	gross hematuria	-	-	Right upper pole	aneurysmal	embolization	-
3	31/F	gross hematuria	-	recent delivery	Right upper pole	cirsoid	embolization	-
4	34/F	gross hematuria	-	recent delivery	Left upper, mid and lower pole	cirsoid	nephrectomy	-
5	37/F	gross hematuria	-	-	Right mid portion	aneurysmal	embolization	+(nephrectomy)
6	50/F	microscopic hematuria	-	-	Left upper pole	cirsoid	embolization	-
7	52/F	gross hematuria	-	-	Right lower pole	cirsoid	embolization	-
8	31/F	gross hematuria	--	+	Right mid portion	aneurysmal	embolization	-
9	38/F	gross hematuria	-	-	Right lower pole	cirsoid	embolization	-
10	40/F	gross hematuria	-	-	Right lower pole	cirsoid	embolization	-
11	39/F	gross hematuria	-	-	Left mid pole	cirsoid	embolization	-
12	26/F	gross hematuria	-	-	Left mid pole	cirsoid	embolization	-
13	32/F	gross hematuria	-	+	Right lower pole	cirsoid	embolization	-

## Data Availability

The data presented in this study are available on request from the corresponding author.
